# Predicting COVID-19 mortality with electronic medical records

**DOI:** 10.1038/s41746-021-00383-x

**Published:** 2021-02-04

**Authors:** Hossein Estiri, Zachary H. Strasser, Jeffy G. Klann, Pourandokht Naseri, Kavishwar B. Wagholikar, Shawn N. Murphy

**Affiliations:** 1 Laboratory of Computer Science, Massachusetts General Hospital, Boston, MA 02144 USA; 2 Department of Medicine, Massachusetts General Hospital, Boston, MA 02144 USA; 3 Harvard Medical School, Boston, MA 02115 USA; 4 Department of Biomedical Informatics, Harvard Medical School, Boston, MA 02115 USA; 5 School of Physics, University of Sydney, Sydney, NSW 2006 Australia; 6 Department of Neurology, Massachusetts General Hospital, Boston, MA 02114 USA

**Keywords:** Statistics, Risk factors, Information technology, Computational science

## Abstract

This study aims to predict death after COVID-19 using only the past medical information routinely collected in electronic health records (EHRs) and to understand the differences in risk factors across age groups. Combining computational methods and clinical expertise, we curated clusters that represent 46 clinical conditions as potential risk factors for death after a COVID-19 infection. We trained age-stratified generalized linear models (GLMs) with component-wise gradient boosting to predict the probability of death based on what we know from the patients before they contracted the virus. Despite only relying on previously documented demographics and comorbidities, our models demonstrated similar performance to other prognostic models that require an assortment of symptoms, laboratory values, and images at the time of diagnosis or during the course of the illness. In general, we found age as the most important predictor of mortality in COVID-19 patients. A history of pneumonia, which is rarely asked in typical epidemiology studies, was one of the most important risk factors for predicting COVID-19 mortality. A history of diabetes with complications and cancer (breast and prostate) were notable risk factors for patients between the ages of 45 and 65 years. In patients aged 65–85 years, diseases that affect the pulmonary system, including interstitial lung disease, chronic obstructive pulmonary disease, lung cancer, and a smoking history, were important for predicting mortality. The ability to compute precise individual-level risk scores exclusively based on the EHR is crucial for effectively allocating and distributing resources, such as prioritizing vaccination among the general population.

## Introduction

Coronavirus disease 2019 (COVID-19) has wreaked unprecedented havoc on economies and health, with unique manifestations that have been difficult to manage with existing treatments. Yet, it is a disease that has appeared after many millions of dollars invested in the worldwide informatics infrastructure. The greater availability of electronic health record (EHR) data has enabled an explosion of studies on COVID-19. At an extremely rapid pace, hospital systems have been developing COVID-19 data repositories to track patient data and try to discover risk factors and treatments for the disease. Standard terminologies have been expanded to include new diagnosis codes and laboratory tests to make the disease analyzable by statistics and machine learning algorithms. In just 3 months, tens of thousands of COVID-19-related studies were published. Early studies were primarily population descriptions, but more recent work has used hospital data to identify risk factors^1–10^.

Many of the data-driven studies do not take full advantage of the richness of EHRs. Many isolate a small subset of EHR data to study only COVID-19 hospitalizations. Few studies use the entire longitudinal medical records. This is likely because EHRs are rife with missing and miscoded data, making analysis potentially misguided by confounders and overfitting. EHR data may not perfectly suit the definition and scope of classic epidemiological studies. Yet, the sheer vastness of the EHR is also an advantage: by using the full medical records, it becomes possible to perform predictive analytics, simply because there is so much data. This possibility is more prominent during pandemics, when access to large-scale research-grade data are not feasible.

Mass General Brigham (MGB) started the COVID-19 Center of Excellence, of which one pillar is analytics. For this, our team developed the COVID-19 datamart, a frequently refreshed snapshot of longitudinal data on patients with a COVID-19 infection flag from many data sources from across the enterprise. This resource provides a system-wide opportunity to perform COVID-related analytics.

We utilized the MGB COVID-19 datamart containing longitudinal medical records from 16,709 COVID-19 patients, to predict risk of mortality and study risk factors for death across different age groups. We demonstrate that the data already routinely collected and stored in EHRs can be rapidly leveraged to address pressing questions related to the COVID-19 pandemic. The initial models we trained by May 2020 (<3 months after the COVID-19 surge in the United States) achieved comparable predictive power to the models described in this study (which were trained in October 2020). Applying a computational algorithm, Minimize Sparsity, Maximize Relevance (MSMR)^11,12^, enhanced with clinical expertise, we constructed clusters of mortality predictors from EHR data for COVID-19 patients. Using these covariates, we developed a set of nested generalized linear models (GLMs) to predict mortality and understand the relationship of various demographics and diseases with COVID-19 mortality. The predictive model provided the ability to forecast the most severe COVID-19 outcome (i.e., death) based on past medical records, which is of special importance for managing hospital resources and making preventive policies, as the virus continues to threaten our wellbeing. We controlled for the confounding effects of age by stratifying models into age-separated cohorts. As most known risk factors for COVID-19 mortality are correlated with age, this stratification allowed us to study the relative importance of different risk factors for each of the age groups.

To date, a number of COVID-19 epidemiology studies have identified risk factors associated with mortality. Several of the risk factors are based on specific demographics. Initial studies from China showed significant increased risk of mortality among patients older than 65 with COVID-19^1–4^. Race and ethnicity have also been identified as potential risk factors. Data collected from the health departments of Chicago and New York City showed increased mortality per 100,000 people in Black and Latinx patients^5,6^. The Centers for Disease Control and Prevention (CDC) also reported disproportionately elevated levels of mortality among Black and Latinx patients based on death certificates^7,8^. Sex has also been identified as a risk factor. In both China and the United States, more men than women were hospitalized with COVID-19^8,9^. The meta-analysis by Jin et al.^10^ showed significant differences in mortality between men and women with the disease.

Patient comorbidities have also been associated with increased risk for severe outcomes. Although in Wuhan, 42.6% of hospitalized patients with COVID-19 had at least one comorbidity^13^, in the United States the CDC reported that 89% of hospitalized patients with COVID-19 had at least one comorbidity^8^. The retrospective cohort study by Zhou et al.^3^ identified a number of comorbidities with significant differences in prevalence between survivors and non-survivors. This included hypertension (HTN), diabetes mellitus (DM), coronary artery disease (CAD), chronic obstructive pulmonary disease (COPD), and chronic kidney disease (CKD)^3^. The retrospective study by Wu et al.^1^ reported a significant difference between the rates of HTN and diabetes in those who developed acute respiratory distress syndrome among COVID-19 patients. Beyond demographics and comorbidities, specific symptoms (i.e., dyspnea), biomarkers (i.e., elevated ferritin, d-dimer, high-sensitivity C-reactive protein), and imaging (abnormalities on chest X-ray image) have also been associated with increased mortality in COVID-19^1–3,13,14^.

In addition, many models have been developed that sought to predict COVID-19 outcomes. Wynants et al.^15^ systematically reviewed and critically appraised 145 prediction models for COVID-19, which included many pre-print manuscripts that were not peer-reviewed. Wynants et al.^15^ labeled all 50 models that prognosticate COVID-19 severity as “poorly reported, highly biased, and overly optimistic.” Of those 50 models, 7 were published in peer-reviewed journals, 4 of which reported the area under the reciever operating curve (AUC-ROC) values for their model. This included the retrospective analysis by Yuan et al.^16^ of 27 COVID-19 patients, which used a scoring system for computed tomography (CT) scans that had previously predicted avian influenza mortality. Yuan’s CT score predicted mortality with an AUC-ROC of 0.90 (95% confidence interval (95% CI): 0.87, 0.93). Colombi et al.^17^ examined 236 patients with COVID-19 and found that including results of CT scans of the chest together with clinical findings increased their AUC-ROC from 0.83 (95% CI: 0.78–0.88) to 0.86 (95% CI: 0.81–0.90). Gong et al.^18^ created a model based on 189 COVID-19 patients that included biomarkers to identify likelihood of severe disease. Gong’s model had an AUC-ROC of 0.85 (95% CI: 0.79–0.92). Finally, Hu et al.^19^ examined how different prognostic scores that included vital signs could predict mortality. Depending on the model used, the AUC-ROC of Hu et al.^19^ ranged from 0.68 (95% CI: 0.58–0.77) to 0.84 (95% CI: 0.76 to 0.91). Together, these models have a wide range of AUC-ROC values from 0.68 to 0.90. However, they all relied on features that were collected at the time of diagnosis, including vital signs, symptoms, biomarkers, and/or images, whereas our model relied exclusively on previous medical records, which could offer advantages by predicting who in our society would be at greatest risk if they were to contract the virus.

## Results

### Features

We began with over 53,100 distinct initial features (i.e., diagnosis and medication records) available in the study population’s EHRs. The first step of the MSMR algorithm reduced the dimensionality to under 9500 distinct features, which we then reduced to 1000 using the joint mutual information (JMI) score. Using clinical expertise, we grouped these medical records into 46 clusters of prior clinical conditions, to which we added other available relevant diagnosis or medication records in the MGB EHR repository. These clusters represent pseudo-cohorts of diseases and we used them as covariates for mortality after COVID-19. For example, the pseudo-cohort of CAD patients encompassed COVID-19-positive patients who had International Classification of Diseases, 10th Revisions (ICD-10) codes such as “Diagnosis of Old Myocardial Infarction” and “Atherosclerotic Heart Disease of Native Coronary Artery.” In certain cases, if a medication was specifically associated with a disease, the medication code(s) was used to identify a disease cluster. For example, the patients taking levothyroxine were designated as having hypothyroidism. Table 1 also includes the list of the 46 clusters of prior conditions. Certain disease pseudo-cohorts were subdivided into two clusters between a non-severe and severe subtype. For example, HTN was divided into a category of HTN and urgent/emergent HTN. The final 46 clusters included all major and subdivided clusters.

**Table 1:** Univariate analysis of the 51 covariates (46 clinical + 5 demographic).

Covariates	Survivors (*N* = 15,879) no. (%)	Non-survivors (*N* = 830) no. (%)	*p*-Value	Relative risk	95% CI		Odds ratio	95% CI	
Female	9168 (58.1)	391 (47.1)	<0.001	0.67	0.58	0.76	0.65	0.57	0.75
Hispanic	1567 (9.9)	27 (3.3)	<0.001	0.32	0.22	0.47	0.31	0.20	0.45
White	8374 (53)	608 (73.3)	<0.001	2.36	2.03	2.74	2.45	2.10	2.88
Black or African American	2326 (14.7)	104 (12.5)	0.102	0.84	0.69	1.03	0.84	0.67	1.03
Under 45 years	6903 (43.7)	21 (2.5)	<0.001	0.04	0.02	0.06	0.03	0.02	0.05
45–65 Years	5629 (35.7)	142 (17.1)	<0.001	0.39	0.33	0.47	0.38	0.31	0.45
65–85 Years	2659 (16.8)	385 (46.4)	<0.001	3.88	3.41	4.43	4.30	3.73	4.96
Over 85 years	688 (4.4)	282 (34.0)	<0.001	8.35	7.35	9.49	11.36	9.65	13.36
Abdominal aortic aneurysm	110 (0.7)	41 (4.9)	<0.001	5.70	4.35	7.46	7.47	5.12	10.68
Atrial fibrillation and flutter	996 (6.3)	266 (32.0)	<0.001	5.77	5.05	6.60	7.05	6.00	8.26
Anemia	4485 (28.4)	489 (58.9)	<0.001	3.38	2.96	3.87	3.64	3.16	4.20
Aortic valve disorder	557 (3.5)	170 (20.5)	<0.001	5.66	4.87	6.59	7.09	5.86	8.54
Benign prostate hypertrophy	1552 (9.8)	255 (30.7)	<0.001	3.66	3.18	4.20	4.09	3.50	4.78
Coronary artery disease	1577 (10.0)	347 (41.8)	<0.001	5.52	4.85	6.28	6.51	5.62	7.55
Cardiomegaly	739 (4.7)	154 (18.6)	<0.001	4.03	3.43	4.74	4.67	3.85	5.63
Chronic kidney disease	1212 (7.7)	342 (41.2)	<0.001	6.83	6.01	7.77	8.48	7.29	9.85
Chronic obstructive pulmonary disease	731 (4.6)	179 (21.6)	<0.001	4.77	4.10	5.55	5.70	4.74	6.82
Cerebrovascular accident	1082 (6.9)	255 (30.7)	<0.001	5.10	4.45	5.84	6.07	5.16	7.11
Depression	4471 (28.3)	356 (42.9)	<0.001	1.85	1.62	2.11	1.92	1.66	2.21
Diverticulosis	1431 (9.1)	177 (21.6)	<0.001	2.55	2.17	2.98	2.74	2.29	3.25
Diabetes mellitus, type 1	503 (3.2)	87 (10.5)	<0.001	3.20	2.60	3.93	3.58	2.80	4.53
Diabetes mellitus, type 2, with complications	1789 (11.3)	293 (35.3)	<0.001	3.83	3.35	4.39	4.30	3.69	4.99
Diabetes mellitus, type 2, without complications	2658 (16.8)	360 (43.4)	<0.001	3.47	3.05	3.96	3.81	3.30	4.40
Epilepsy	551 (3.5)	72 (8.7)	<0.001	2.45	1.95	3.08	2.65	2.03	3.40
End-stage renal disease	300 (1.9)	83 (10.0)	<0.001	4.74	3.87	5.80	5.78	4.46	7.41
Gastroesophageal reflux disease	3429 (21.7)	286 (34.5)	<0.001	1.84	1.60	2.11	1.91	1.64	2.21
Gastrointestinal bleed	1339 (8.5)	183 (22.0)	<0.001	2.82	2.42	3.30	3.07	2.58	3.65
Gout	553 (3.5)	117 (14.1)	<0.001	3.93	3.28	4.70	4.55	3.66	5.61
Heart failure	1081 (6.8)	301 (36.3)	<0.001	6.31	5.54	7.19	7.79	6.67	9.08
Hyperlipidemia	5170 (32.7)	563 (67.8)	<0.001	4.04	3.50	4.65	4.37	3.76	5.08
Hypertension	5867 (37.2)	672 (81.0)	<0.001	6.61	5.58	7.84	7.25	6.10	8.68
Hypertensive emergency	289 (1.8)	64 (7.7)	<0.001	3.87	3.07	4.88	4.51	3.38	5.94
History of pneumonia	2940 (18.6)	406 (48.9)	<0.001	3.82	3.36	4.36	4.21	3.66	4.86
History of a urinary tract infection	3607 (22.8)	369 (44.5)	<0.001	2.56	2.25	2.93	2.72	2.36	3.14
Hyperparathyroidism	342 (2.2)	74 (8.9)	<0.001	3.83	3.08	4.77	4.45	3.40	5.75
Hypothyroidism	1635 (10.4)	180 (21.7)	<0.001	2.27	1.94	2.66	2.41	2.03	2.86
Interstitial pulmonary disease	207 (1.3)	53 (6.4)	<0.001	4.32	3.36	5.54	5.17	3.76	7.00
Mitral valve disorder	945 (6.0)	186 (22.4)	<0.001	3.98	3.42	4.63	4.57	3.82	5.43
Breast neoplasm	356 (2.3)	51 (6.1)	<0.001	2.62	2.01	3.42	2.86	2.09	3.84
Lung neoplasm	147 (0.9)	43 (5.2)	<0.001	4.75	3.62	6.23	5.86	4.09	8.22
Prostate neoplasm	260 (1.6)	54 (6.5)	<0.001	3.63	2.82	4.68	4.19	3.07	5.62
Osteoarthritis	3307 (20.9)	369 (44.5)	<0.001	2.84	2.49	3.24	3.04	2.64	3.51
Occlusion of the carotid artery	443 (2.8)	130 (15.7)	<0.001	5.23	4.42	6.18	6.47	5.23	7.96
Obstructive sleep apnea	1529 (9.7)	128 (15.4)	<0.001	2.84	2.49	3.24	3.04	2.64	3.51
Parkinson’s disease	133 (0.8)	32 (3.9)	<0.001	4.02	2.92	5.53	4.76	3.16	6.96
Pulmonary embolism	795 (5.0)	145 (17.5)	<0.001	3.55	3.01	4.19	4.02	3.30	4.86
Pulmonary hypertension	249 (1.6)	73 (8.8)	<0.001	4.91	3.96	6.07	6.06	4.59	7.91
Peripheral vascular disease	529 (3.4)	145 (17.5)	<0.001	5.04	4.28	5.92	6.15	5.02	7.48
Rheumatoid arthritis	351 (2.2)	45 (5.4)	<0.001	2.36	1.78	3.14	2.54	1.83	3.46
Smoking history	1304 (8.3)	137 (16.5)	<0.001	2.09	1.76	2.50	2.21	1.82	2.67
Thoracic aortic aneurysm	177 (1.1)	35 (4.2)	<0.001	3.43	2.51	4.67	3.92	2.67	5.60
Tricuspid valve disorder	569 (3.6)	126 (15.2)	<0.001	4.12	3.47	4.91	4.82	3.90	5.91
Vitamin D deficiency	2244 (14.2)	164 (19.8)	<0.001	1.46	1.24	1.73	1.50	1.25	1.78
Ventricular tachycardia	249 (1.6)	82 (9.9)	<0.001	5.42	4.44	6.63	6.89	5.28	8.90

Difference in the mortality probabilities between the group with the condition (or demographic status) and the no-condition group.

The list of diagnosis and medication codes used to curate disease clusters are provided in Supplementary Table 1. In addition to these features, we included five demographic covariates, including age (in years), and binary variables to describe gender (female), race (Black and White), and ethnicity (Hispanic). Overall, 51 covariates were used for the analysis.

### Univariate analysis

The overall probability of death in our COVID-19 patients was 4.9%. Twenty-five percent of the patients in this cohort were hospitalized. The hospitalized patients encompassed 77% of the 830 death events. Table 1 shows that probability of death fluctuated by demographics. Of the measured demographics, the two groups of under 45 years and between 45 and 65 years were both associated with a decreased risk of mortality. The odds ratio (OR) for mortality in those under 45 years was 0.034 (CI: 0.021–0.051). Those between 45 and 65 years had an OR for mortality of 0.38 (CI: 0.31–0.45). Being identified as Hispanic or female was also associated with a decreased risk of mortality with an OR of 0.31 (CI: 0.20–0.45) and 0.65 (CI: 0.57–0.75), respectively. Identification as Black was not found to be statistically significant, whereas being identified as White was associated with increased mortality with an OR of 2.45 (CI: 2.10–2.88). Finally, ages between 65–85 years and greater than 85 years were both associated with increased probability of mortality with an OR of 4.30 (CI: 3.73–4.96) and 11.36 (CI 9.65–13.36), respectively.

All of the comorbidities were associated with increased risk of mortality; hence, their ORs are all >1. The five comorbidities with the highest ORs were CKD, heart failure, abdominal aortic aneurysm, HTN, and aortic valve disease.

### Model comparison

The overall model, which included patients under 45 years, had a mean AUC-ROC of 0.898 with a 95% CI between 0.896 and 0.900 (Table 2).

**Table 2:** The mean area under the operating characteristics curve (AUC-ROC) by model.

Model	GLM w/ gradient boosting	Standard GLM	*p*-Value^a^
Overall model	0.898 [0.896, 0.900]^b^	0. 892 [0.889, 0.896]	0.0038
45–65 Years	0.789 [0.795, 0.809]	0.688 [0.675,0.701]	0.00001
65–85 Years	0.753 [0.745,0.760]	0.715 [0.705,0.725]	0.00001
85+ Years	0.685 [0.673, 0.697]	0.641 [0.626,0.656]	0.0001

^a^*p*-Values from the Wilcoxon’s rank-sum test.

^b^95% confidence intervals in brackets.

**Fig. 1:**
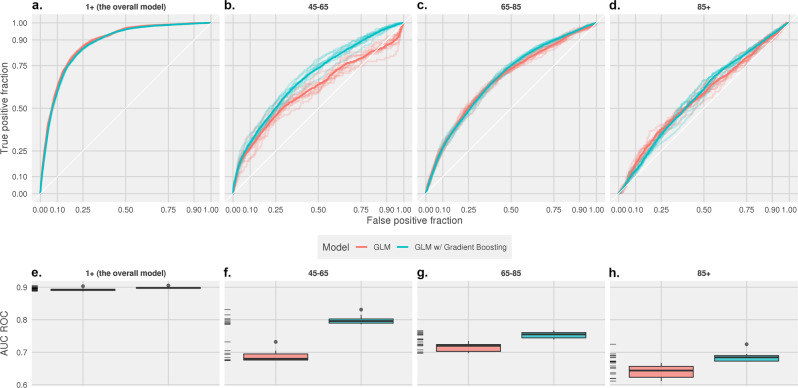
Comparing the classification performance between the boosting and standard GLM (binomial logistic) models. **a**–**d** The ROC curves for the overall 45–65, 65–85, and 85+ models, respectively. **e**–**h** The area under the ROC curves for the overall, 45–65, 65–85, and 85+ models.

Figure 1 illustrates the difference in prediction performances (ROC curves and AUC ROCs) between the standard GLM and boosting GLM, both with the logit link and binomial distribution. In the overall model, a Wilcoxon’s rank-sum test was performed to compare the standard GLM with the boosting GLM (mean AUC ROCs 0.892 [CI: 0.889–0.896] vs. 0.898 [CI: 0.896–0.900]). Although there was a small absolute difference between the two models (a difference of 0.005), the *p*-value was significant at 0.0038. However, the boosting algorithm offered an even greater improvement to the age-based models. In the 45–65 cohort, the boosting algorithm improved the median prediction performance measure (AUC-ROC) computed on the held-out set by 15% (0.789 vs. 0.688). The improvements in prediction provided by the boosting algorithm were over 5% in the 65–85 cohort (0.753 vs. 0.715) and 7% in the 85+ cohort (0.685 vs. 0.641). Each of the models was compared to one another with a Wilcoxon’s rank-sum test and showed a *p*-value < %.

**Fig. 2:**
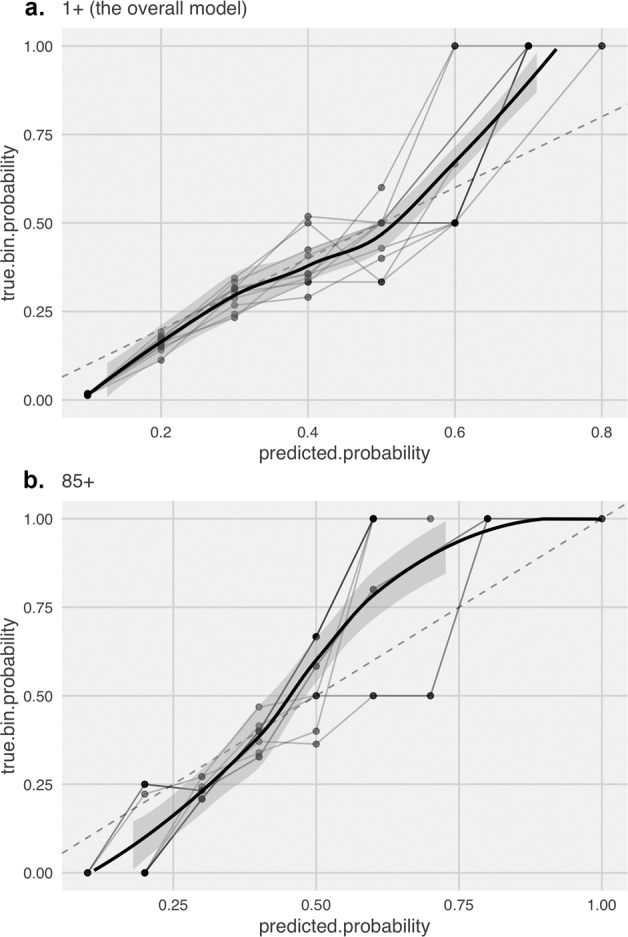
Calibration curves of models. **a** The calibration curve for the overal model. **b** The calibration curve for the 85+ model.

We also plotted the diagnostic reliability diagrams (calibration curves) to test the reliability of the models and compare the algorithms. Figure 2 illustrates the calibration curves for the overall and 85+ models. To compare the two algorithms, we fitted a smoothed trend line as a representation of the overall calibration curve obtained from the ten calibration plots for each algorithm. The calibration plots are produced from the raw predicted probabilities computed by each algorithm (*X* axis) against the true probabilities of patients falling under probability bins (*Y* axis). In a well-calibrated model, the calibration curve appears along the main diagonal—the closer the more reliable. As illustrated in Fig. 2, the calibration curves from the boosting algorithm are close to the diagonal, which means that the models are generally reliable. Despite the boosting algorithm not having an acceptable discrimination power for the oldest age group, the calibration curve demonstrates relatively reliable probabilities.

### Overall model

**Fig. 3:**
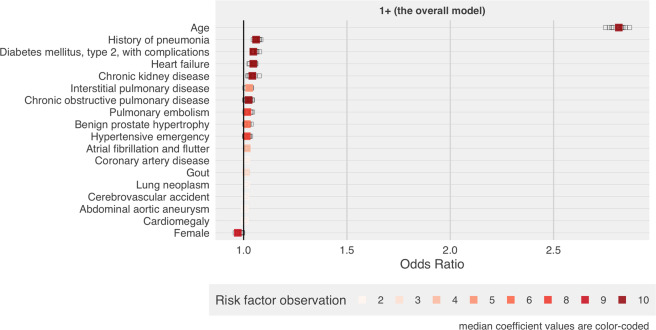
Odds ratios for the covariates identified as predictors of mortality in COVID-19 patients. Risk factor observation represents the number of model iterations that identified a covariate as a predictor of mortality in COVID-19 patients. The total number of model iterations is 10. Median and interquartile ranges (IQR) for odds ratios are available in Supplementary Table 2.

In the overall model, 18 features were identified in at least 20% of the models (2 of 10) as being associated with an increased mortality risk (Fig. 3). We present Odds Ratios (ORs) with interquartile ranges (IQR) to compare relative importance of the variables for predicting mortality. Of these features, age had the most prominent association—median OR: 2.82 (iqr: 0.03)—for predicting mortality. All median ORs are provided in Supplementary Table 2. It is important to note that the ORs from the boosting models might be smaller than those obtained from a standard model, as boosting has the effect of shrinkage. The coefficient for the next highest feature was considerably smaller. We found that several respiratory diseases were associated with an increased mortality risk including a history of pneumonia (OR: 1.06, iqr: 0.02), COPD (OR: 1.02, iqr: 0.02), interstitial pulmonary disease (OR: 1.03, iqr: 0.02), and a history of a pulmonary embolism (OR: 1.02, iqr: 0.02). Other identified risk factors included diseases of the heart and vascular system such as a history of cardiomegaly, hypertensive urgency or emergency, heart failure, cardiomegaly, and atrial fibrillation or flutter. In addition, diseases affecting diverse organs were associated with various degrees of risk in the model including a history of a cerebrovascular accident, CKD, diabetes with complications, benign prostatic hypertrophy, and gout.

### Age-based models

**Fig. 4:**
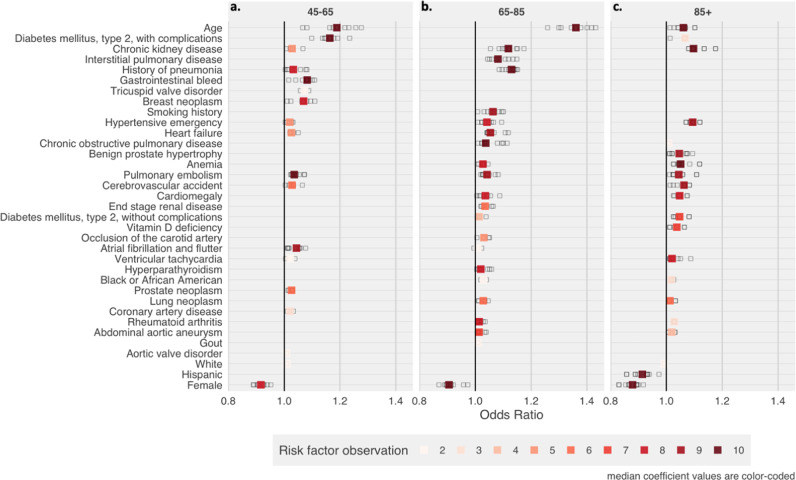
Odds ratios for the covariates identified as predictors of mortality in COVID-19 patients by age groups. **a** Odds ratios for the 45-65 model. **b** Odds ratios for the 65-85 model. **c** Odds ratios for the 85+ model. Risk factor observation represents the number of model iterations that identified a covariate as a predictor of mortality in COVID-19 patients. The total number of model iterations is 10.

As described in the methods, we stratified the overall model by age groups in order to reduce the variability in age and to identify risk factors of different ages. We created three age groups (Fig. 4) in which age was fixed to a 20-year variation: 45 to 65, 65 to 85, and above 85 years (the oldest patient group included at least a 20-year age variation). As presented in Table 2, we found that the prediction performance declined from an AUC-ROC of 0.898 in the overall group to between 0.685 and 0.789 for the different age groups.

Among the 45–65-year age group, there were 17 features associated with increased mortality. Many of these features were not only associated with the pulmonary system, but included diseases affecting various organs. The highest risk disease clusters were a history of diabetes with complications (OR: 1.16, iqr: 0.03), a gastrointestinal bleed (OR: 1.08, iqr: 0.05), breast cancer (OR: 1.07, iqr: 0.03), and a tricuspid valve disorder (OR: 1.07; iqr: 0.01). Disorders unique to this specific age cohort included atrial fibrillation and atrial flutter, CAD, and tricuspid valve disorders.

Among the 65–85-year age group, 21 features were associated with increased risk. For this group, many of the features were related to the respiratory system. A history of pneumonia (OR: 1.13, iqr: 0.03), interstitial pulmonary disease (OR: 1.08, iqr: 0.05), COPD (OR: 1.04, iqr: 0.07), pulmonary embolism (OR: 1.04, iqr: 0.02), and lung cancer (OR: 1.03, iqr: 0.02) were all associated with increased risk of mortality. Smoking (OR: 1.06, iqr: 0.04) was also associated with an increased risk. A few features were associated with the cardiac and vascular system including a history of cardiomegaly, heart failure, atrial fibrillation, and hypertensive urgency and emergency. Unique features for this age group included a smoking history and occlusion of the carotid artery.

In the 85+ years age group, there were 17 features associated with increased mortality. The highest risk disease clusters included CKD (OR: 1.10, iqr: 0.04), hypertensive emergency (OR: 1.09, iqr: 0.01), cerebrovascular accident (OR: 1.06, iqr: 0.03), and DM type 2 with complications (OR: 1.06, iqr: 0.03). There were several features exclusive to this model including Vitamin D deficiency and benign prostate hypertrophy.

### Demographic covariates

Among the demographic features, age was the most important predictor of mortality in the overall COVID-19 patient population. However, in the age-based models where the variability in age was limited to 20 years, we found its association diminish from a median OR of 2.82 in the overall model to 1.19 in the 45–65 model, to 1.36 in the 65–85 model, and 1.06 in the patients over 85 years. Despite separating cohorts into different ages, the age feature continued to be an important predictive factor in mortality within those groups. This finding was most apparent among the 65–85-year-old age group.

For gender, we found that being female consistently reduced the risk of death—the median OR in the overall model was 0.98 (iqr: 0.005). In each of the age models, by limiting the age range and thereby reducing the importance of age, being female became more strongly associated with decreased mortality.

In the overall model, we did not find any evidence that a certain race or ethnicity would alter the chance of mortality after COVID-19. However, among the oldest cohort of patients, we found that being Black (or African American) was associated with a higher chance of mortality and being Hispanic or White was associated with a lower risk.

## Discussion

We demonstrated that EHR data can be rapidly leveraged for predicting outcomes and studying newly emerging infectious diseases. We were able to train early versions of the models presented in this paper by May 2020 with similar predictive power, only 3 months after the COVID-19 pandemic was recognized in the United States. The models developed in this study leveraged only the demographics, diagnoses, and medications, in order to predict who, with a COVID-19 diagnosis, was at risk of death. Even though the features independently have relatively low ORs (see Table 1), our overall model still has an AUC-ROC of 0.898. Other published COVID-19 predictive models for mortality have AUC-ROC’s that range from 0.68 to 0.90^16–19^, relying on an assortment of symptoms, laboratory values, and images at the time of diagnosis or during the illness to predict mortality.

Our model was able to provide a relatively high AUC-ROC, while relying exclusively on findings already stored in the EHR. For each patient, the model predicted a probability for death, solely based on the past medical records. We demonstrated the clinical reliability of the models through calibration curves. This has substantial implications, especially as the virus continues to spread rapidly. At the point of care, the estimated probabilities can be used to construct risk strata to quickly separate low risk patients from those with a high risk of mortality. This technique could be extremely valuable for determining who is most likely to benefit when resources are limited, such as informing vaccination distribution. Currently, the CDC recommendations for vaccinations rely on generalizations that are based on age, occupation, and the living environment^20^. Such recommendations are imprecise and do not leverage the wealth of information in the EHR. A model, such as the one proposed in this study, could assess the individual risk of every participant in a healthcare system based off of demographics, diagnoses, and medications found in the EHR. This could ensure resources are being equitably distributed by identifying and then prioritizing the highest risk individuals in each of the groups.

Our results show that the boosting algorithm improved the discrimination power in age-based models over the standard GLM algorithm by 5–15%. Although previous COVID-19 epidemiology studies have identified chronic diseases that independently have an impact on prognosis, many of the risk factors identified were highly correlated with one another and therefore when combined do not necessarily lead to a significantly higher likelihood of an adverse outcome. For example, in our overall model, HTN had a correlation with age of 0.36, with hyperlipidemia of 0.67, and with DM type 2 with complications of 0.41. Although early research findings observed higher rates of HTN among severely ill COVID-19 patients^21–23^, in the case of HTN, it is not clear if the disease actually caused a higher mortality or if it is a confounder. Our model provides a framework for understanding how multiple risk factors can collectively be interpreted to predict death after a COVID-19 diagnosis. Of note, certain risk factors like HTN were not included in our final model.

Despite certain features dropping out, the features that were ultimately included in the overall model correspond well with previously identified risk factors from previous epidemiology studies. For example, in our overall model, age was by far the most important feature for predicting COVD-19 mortality. Previous epidemiology studies have shown higher rates of case fatalities among older patients^3,24^. This model demonstrated just how significant the age feature (OR of 2.82) is compared to the next highest feature of pneumonia (OR of 1.06). In addition, our overall model supported the previous epidemiological findings that women with COVID-19 have an overall lower chance of death compared to men^5–9^. Our overall model, associated the female label with having an OR of 0.972. Further, this feature was present in nearly every fold of the overall model (9 out of 10).

Several features identified in the models warrant further evaluation. The second most important feature in the overall model was a history of pneumonia. This feature also appeared in some of the age group models as well. Many of the COVID-19 epidemiology studies do not report a history of pneumonia as a risk factor^1,3,8^. However, the MSMR method of selecting features from the totality of longitudinal data of a patient’s EHR led to its identification. Its incorporation into the final prediction model was due to it not only being predictive, but likely less correlated with many other chronic diseases like HTN, hyperlipidemia, CAD, and age for predicting death. It is possible that the diagnostic label was a proxy for an underlying chronic lung disease. These findings suggest that future studies should investigate this correlation between a previous pneumonia diagnosis and increased risk of death with COVID-19.

The model also identified several features that were not specifically seen in the initial COVID-19 epidemiology studies. In the initial study in China by Zhou et al.^3^, only 2 of the 191 patients had CKD^3^. Subsequent studies such as that of Cheng et al.^13^ have shown significant increased mortality in patients with acute kidney injury at the time of COVID-19 diagnosis, but did not comment specifically on previous CKD. Our overall model showed that CKD was one of the more important risk factors for assessing the potential for death due to COVID-19 in the overall population with an OR of 1.04 in the overall model. Among the 85+ years age group, it is one of the most important features for predicting mortality.

Interestingly, the top features of the overall model span a number of different organ systems. After age, the top features were a history of pneumonia, CKD, DM type 2 with complications, and heart failure. These diseases correspond to the respiratory, renal, endocrine, and cardiovascular systems, respectively. The findings suggest that simply adding the commonly identified risk factors such HTN, diabetes, and CAD will not lead to the most predictive model. Rather there is an increased risk specifically for patients that have a diverse set of comorbidities spanning different organ systems.

When the models were separated into different age groups, age became less of a prominent feature compared to its effect in the overall model. Still, even among the 45–65-year-old patients, of all the features included in the model, age remained the most important predictor for death. In addition, in this younger age group, the next most important feature was a history of DM type II with complications. The model showed that when evaluating risk among 45–65-year-olds, the most important features were the relative age within the group and a history of DM with complications.

Among the 65–85-year-olds, age remained the most important feature for predicting death among COVID-19 patients. In this age range, many respiratory diseases independently increased the risk for COVID-19 mortality. This included a history of pneumonia, COPD, lung cancer, pulmonary embolism, interstitial pulmonary disease, and smoking. Despite these diseases all being related to the respiratory system, each individually still contributed to an increased risk of mortality among COVID-19 patients. They did not have substantial correlation with one another despite all of them having an effect on the lungs. When assessing patients in this age range, the model suggests that it could be helpful to quantify the number of chronic diseases affecting the lung.

Unlike other studies that began with a limited set of hypothetical risk factors, we took a primarily inductive approach for identifying potential COVID-19 risk factors. This approach allowed us to filter through thousands of medical records to identify potential risk factors for death after COVID-19, some of which, like the history of pneumonia, might have never been included in an epidemiological study with covariates chosen a priori.

Finally, our initial univariate analysis showed an increased risk of mortality among patients who identified as White, no difference among those who identified as Black, and a decreased risk among those who identified as Hispanic. After taking into account demographics, diagnoses, and medications, the risk of death associated with each race and ethnicity was no longer evident in the overall model. In the oldest age model Black patients had an increased risk and Hispanic and White patients had a decreased risk. Every machine learning model is subject to the constraints of the specific population that it trains on. Although our model may have been trained on a dataset that had a high association of White patients with comorbidities, it accounted for this in the adjusted model and no longer showed the association in the actual model. The findings of Black patients having an increased risk of mortality corresponds with previous epidemiology studies that have identified increased risk among minorities^7,8^.

This study relied on retrospective data from the EHRs. As a result, there are considerations that need to be taken into account when analyzing the data. First, the history of a medical record does not guarantee that the patient currently has (or maybe ever had) the respective clinical condition. It could be that a disease was resolved over time or never even existed. Second, multiple imputation for missing diagnosis and medication records was not possible. As a result, if a patient did not have any EHR from a disease cluster, we assumed that the history of disease was not present. Third, our models did not fully control for all confounders, which could bias some of the findings. For example, high healthcare utilization among patients may lead to artificially increased numbers of diagnoses, compared to patients with low healthcare utilization. Future studies should evaluate specific features by attempting to control for confounders. The risk factors identified in this study should be understood collectively as a powerful tool for ascertaining risk in COVID-19 patients. Finally, external validation of the predictive models and features in this study should eventually be tested on data from other healthcare institutions.

The ever-increasing availability and prevalence of EHR systems offer longitudinal medical data on millions of patients. EHR data can be rapidly leveraged for predicting outcomes and studying pandemics. We trained our initial models with comparable metrics in May 2020, roughly 3 months after the COVID-19 pandemic was recognized in the United States. These data are not clean and may not perfectly suit the definition and scope of classic epidemiological studies. However, when coupled with innovative approaches, EHR data can be useful for predicting and understanding disease trajectories and treatment outcomes. This possibility is more prominent during pandemics, when access to large-scale research-grade data is not feasible. We showed that EHR data can be leveraged to predict death after COVID-19. This capability enables us to predict the most severe COVID-19 outcome (i.e., mortality) based exclusively on the past medical records. It is of special importance for allocating finite resources and guiding preventative policies as the virus continues to spread. Inevitably, when new viruses emerge, predictive modeling capabilities with EHR data will be crucial for allocating therapies, vaccinations, and other resources.

## Methods

### Study goals

The primary goal of this study was to create a predictive model for COVID-19 mortality based on the longitudinal data stored in EHRs, in hopes that longer medical history will allow improved predictive performance. We strive to (1) develop a predictive model for the overall population and specific age groups based on longitudinal medical records (as initial features) stored in the EHRs and (2) analyze and interpret the importance of the features identified by each of the models, to see if it conveys some explanatory power in the risk factors of the disease.

### Data

**Fig. 5:**
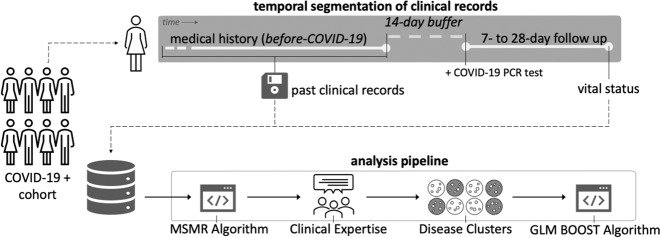
Temporal segmentation of clinical records and analysis pipeline. The analysis pipeline utilizes longitudinal clinical data with a 14-day buffer before COVID-19 infection.

We used EHR’s data from 24,215 patients with a confirmed case for COVID-19 (confirmed polymerase chain reaction test, PCR) between 03 March 2020 and 10 November 2020. We narrowed this cohort to those who had at least 1 year of medical history—i.e., a 1-year time difference between the first and the last medical record before the COVID-19 positive PCR test—with MGB, resulting in a final study cohort of 16,709 patients with a confirmed COVID-19 PCR test. MGB includes 10 hospitals in the Greater Boston area with a total of over 3400 beds, 160,000 discharges per year, and 200 intensive care unit beds. For each patient, we performed temporal data segmentation to eliminate any possible temporal data leakage in the analysis (Fig. 5). We included data from the beginning of the electronic record (as far back as 1 January 2000 for some patients) up to 14 days prior to the positive COVID-19 PCR test date. This temporal buffer ensured that no COVID-19-related medical conditions were included in the model as a risk factor (the study design is illustrated in Fig. 5). Mortality data were retrospectively added to the record from various data sources and this included mortality unrelated to the visit. The use of data for this study was approved by the MGB Institutional Review Board (2020P001063). A waiver of consent was granted due to the rapidly evolving nature of the pandemic, patient intake and visitor policies. Table 1 includes a summary of demographic information about the study cohort.

### Feature selection

Unlike other studies that begin with a limited set of hypothetical risk factors, we took an inductive approach for identifying potential risk factors for COVID-19 mortality. The feature selection began with a computational algorithm that MSMR^11,12^. We enhanced the algorithmic feature selection with clinical knowledge by constructing clusters of past medical conditions from the MSMR’s initial features. Finally, we utilized a boosting algorithm to identify covariates that were associated with the risk of death in COVID-19 patients.

### Minimize Sparsity, Maximize Relevance

The MSMR^11,12^ algorithm is a filter method that aims to minimize sparsity and maximize relevance in a high dimensional feature space. All steps of the MSMR algorithm were performed in R statistical language^25^. MSMR filtered out sparse medical records that were utilized in this study as initial features, removing medical records seen in <0.25% of the patient population (fewer than ~20 patients). MSMR then computed the JMI^26,27^ score for each of the remaining medical records (applying the praznik R package)^28^. JMI represented the mutual information between a set of features and an outcome, while also taking into account the redundancy between the features—i.e., reducing multicollinearity among covariates. Using a combination of JMI score and the observation frequency, MSMR ranked and limited the number of potential initial features (i.e., medical records) to the top *N* features. The top 1000 initial features were used in this study for expert covariate clustering.

### Expert feature clustering

EHR records often contain different concept codes (e.g., codes from the ICD-9 or -10, Clinical Modification, known as ICD-9 or ICD-10 codes), which refer to similar clusters of clinical conditions. We utilized the 1000 initial features (including diagnosis and medication records) provided by the MSMR algorithm as an initial set of indicators for potential covariates that could predict death in COVID-19 patients. Using these medical records, a clinician in our team constructed clusters of records to identify patient pseudo-cohorts with similar prior clinical conditions, which we used as clinical covariates in this study. To increase robustness of the clusters, we include additional diagnosis and medication codes into the construction of clusters that were not initially included in the top 1000 codes by MSMR, which may happen due to the variability in the healthcare utilization processes across time. For example, if multiple ICD codes related to HTN are recommended by the MSMR algorithm, the clinician would create one (or more) cluster(s) of hypertensive patients, using all the codes in the EHR that are used to define HTN cohorts. Each cluster was considered a predictor of mortality in COVID-19 patients.

### Univariate analysis

For each of the clusters of prior conditions, we performed a two-proportion *z*-test between survivors and non-survivors, and calculated the *p*-value. The relative risk and OR were calculated for each demographic and disease cluster to compare patients with and without the condition (or demographic status) of interest.

### Boosting algorithm

To further evaluate the identified candidate clusters of risk factors for mortality after COVID-19 and study their potential fluctuations across age groups, we applied GLMs with component-wise functional gradient boosting, implemented in the R^25^ add-on package mboost^29,30^. Boosting algorithms improve the prediction power of the model by training a sequence of weak models that each compensate for the weaknesses of their predecessor^30–32^. A Wilcoxon’s rank-sum test was performed to compare the GLM with gradient boosting to the standard GLM and determine their AUC-ROC difference was statistically significant. We then leveraged the boosting algorithm to perform a final evaluation of the relative importance of the final features obtained from the MSMR and expert clustering.

### Experimental setting

For classifier training and testing, we used an 80 : 20 ratio (i.e., 80% of the data used for training) with random sampling. We iterated the train-test sampling 10 times to account for possible patient population differences caused by the sampling. To train the models, we performed fivefold cross-validation. To evaluate the models’ discrimination power, we computed the AUC-ROC on the held-out test sets. We also evaluated the models’ reliability for clinical interpretation using diagnostic reliability diagrams (calibration curves). We extracted the GLM boosting regression coefficients with the logit link and binomial distribution to study risk factor importance and fluctuations across age groups. We then performed a nonparametric Wilcoxon’s rank-sum test to compare the GLM with boosting to a traditional GLM.

To control for age as a risk factor, we created uniform 20-year age groups from subsets of the patient population with a focus on patients who were over 45 years old. The age groups included 45–65, 65–85, and above 85 years. We repeated the same model for training and evaluation experiments on each of these age groups as was done on the overall patient population.

Together, using the clinical risk factors and the five demographic covariates (binary variables for female, White, Hispanic, and Black or African American, as well as continuous variable for age) we trained and tested four models as follows: (1) the overall model trained and tested on the entire patient population, (2) a model for patients older than 45 years and younger than 65 years (45–65), (3) a model on patients between 65 and 85 years of age (65–85), and (4) a model on patients older than 85 years (85+). Due to the highly unbalanced data, we under-sampled the alive patients to improve model training. That is, we limited patients to those who survived for at least a specific number of days after contracting COVID-19, depending on the age group model. In the overall, 45–65, and 65–85 models, we specified a 28-day buffer for the alive patients, but in the 85+ model, where death incidents were more balanced, we only required a 7-day buffer to assume a patient’s survival. We computed ORs by exponentiating the regression coefficients obtained from the GLMs to compare relative importance of the variables for predicting mortality within and across age groups. It is important to note that the ORs from the boosting GLM are expected to be smaller than those obtained from a standard GLM, as boosting has a shrinkage effect.

### Reporting summary

Further information on research design is available in the Nature Research Reporting Summary linked to this article.

## Supplementary information

Supplementary Information

Reporting Summary

## Data Availability

Protected Health Information restrictions apply to the availability of the clinical data here, which were used under IRB approval for use only in the current study. As a result, this dataset is not publicly available. Qualified researchers affiliated with the Mass General Brigham (MGB) may apply for access to these data through the MGB Institutional Review Board.

## Code availability

The R package for MSMR algorithm is available at github.com/hestiri/MSMR.
