# Supplementary material for: Predicting COVID-19 mortality with electronic medical records
Source: NPJ Digit Med. 2021 Feb 4;4:15. doi: 10.1038/s41746-021-00383-x (PMC7862405; doi:10.1038/s41746-021-00383-x)
Supplement: Supplementary file 1 — Supplementary Information [file 41746_2021_383_MOESM1_ESM.pdf]

Supplementary Information for Predicting COVID-19 Mortality with Electronic Medical Records

Supplementary Table 1. List and Definition of Prior Condition Clusters

Cluster Name	Definition<br>*Diagnosis Codes:*<br>- *ID9 - International Classification of Disease 9 (ICD9)*<br>- *ICD10 - International Classification*<br>** also used MGB internal legacy codes*<br>*Medication Codes:*<br>- *ERX – Epic medications*<br>- *OMA - OnCall medications*<br>- *LMA – Longitudinal Medical Records medications*
Abdominal aortic aneurysm	Codes that mention an abdominal aneurysm, including ruptured or unruptured subtypes:<br>- ICD9 441.3 – Abdominal aneurysm, ruptured<br>- ICD9 441.4 – Abdominal aneurysm without mention of rupture<br>- ICD10 I71.3 – Abdominal aortic aneurysm, ruptured<br>- ICD10 I71.4 – Abdominal aortic aneurysm, without rupture
Atrial fibrillation and flutter	Codes that mention either atrial fibrillation or atrial flutter (including paroxysmal):<br>- ICD9 427.3x – Atrial Fibrillation and Flutter<br>- ICD10 I48.x – Atrial Fibrillation and Flutter
Anemia	Codes that mention anemia including iron, hereditary, or acquired subtypes:<br>- ICD9 280.x Iron deficiency anemias<br>- ICD9 281.x Other deficiency anemias<br>- ICD9 282.x Hereditary hemolytic anemias<br>- ICD9 283.x Acquired hemolytic anemias<br>- ICD9 285.x Other and unspecified anemias<br>- ICD10 D50.x Iron deficiency anemia<br>- ICD10 D50.x-53.x Nutritional Anemias<br>- ICD10 D55.x-D59.x Hemolytic Anemias<br>- ICD10 D60.x-D64.x Aplastic and other anemias and other bone marrow failure syndrome<br>- ICD10 O90.81 Anemia of puerperium<br>- ICD10 O99.01x Anemia of pregnancy
Aortic valve disorder	Codes that mention aortic valve disorder including insufficiency, regurgitation, stenosis, and biscupid subtypes:<br>- ICD9 424.1 – Aortic valve disorders<br>- ICD10 I35.x – Aortic valve disorder<br>-
Benign prostate hypertrophy	Codes that mention benign prostate hyperplasia and hypertrophy:<br>- ICD9 600.x – Hyperplasia of prostate<br>- ICD10 N40 – Benign prostatic hyperplasia<br>Codes that mention the medications tamsulosin and finasteride (examples listed):

	- ERX 4004089053<br>- OMA XSBJ8<br>- LMA5230
Coronary artery disease	Codes that mention chronic ischemic disease, pathologic findings of coronary circulation images, and coronary artery disease:<br>- ICD9 414.x – Other forms of chronic ischemic heart disease;<br>- ICD10 I25.x – Chronic Ischemic heart disease<br>- ICD10 R93.1 – Diagnosis of Abnormal Findings on diagnostic imaging of heart and coronary circulation<br>Codes that mention the medication sublingual nitroglycerin (examples listed):<br>- ERX 705113<br>- OMA NSPA3
Cardiomegaly	Codes that mention cardiomegaly or left ventricular hypertrophy:<br>- ICD9 429.3 – Cardiomegaly<br>- ICD10 I51.7 – Cardiomegaly
Chronic kidney disease	Codes that mention chronic kidney disease, but not specifically end stage renal disease:<br>- ICD9 585.x – Chronic kidney disease<br>- ICD10 D63.1 – Anemia in chronic kidney disease<br>- ICD10:E08.22 – Diabetes due to underlying disease with chronic kidney disease<br>- ICD10:E09.22 – Drug or chemical induced diabetes with CKD<br>- ICD10 E10.22 – Type 1 Diabetes with CKD<br>- ICD10 E11.22 - Type 2 Diabetes with CKD<br>- ICD10 E13.22 – Other specified diabetes mellitus with CKD<br>- ICD10 I12.x – Hypertensive chronic kidney disease<br>- ICD10 I13.x – Hypertensive heart and chronic kidney disease<br>- ICD10 N18.x – Chronic Kidney Disease
Chronic obstructive pulmonary disease	Codes that mention to chronic airway obstruction or COPD:<br>- ICD9 496 Chronic airway obstruction, not elsewhere classified<br>- ICD10 J44.x COPD
Cerebrovascular accident	Codes that mention occlusion and infarction of the cerebral arteries, cerebrovascular disease, sequelae of cerebrovascular disease, or stroke:<br>- ICD9 434.x – Occlusion of cerebral arteries<br>- ICD9 436 - Acute, but ill-defined, cerebrovascular disease<br>- ICD9 437.x - Other and ill-defined cerebrovascular disease<br>- ICD9 438.x - Late effects of cerebrovascular disease<br>- ICD10 I63.x – Cerebral Infarction<br>- ICD10 I69.x Sequelae of cerebrovascular disease<br>- ICD10 G46.x – Vascular syndromes of brain in cerebrovascular diseases
Depression	Codes that mention dysthymia, depression, or depressed mood:<br>- ICD9 300.4 Dysthymic disorder<br>- ICD9 290.13 Presenile depression

	- ICD9 290.21 Senile dementia with depressive features<br>- ICD9 290.43 Vascular dementia, with depressed mood<br>- ICD9 296.2x Major depressive disorder single episode<br>- ICD9 296.3x Major depressive disorder recurrent episode<br>- ICD9 309.0 Adjustment disorder with depressed mood<br>- ICD9 309.1 Prolonged depressive reaction<br>- ICD9 311 Depressive disorder, not otherwise specified<br>- ICD10 F06.31 Mood disorder due to known physiological condition with depressive features<br>- ICD10 F06.32 Mood disorder due to known physiological condition with major depressive-like episode<br>- ICD10 F32.x Major depressive disorder, single episode<br>- ICD10 F33.x Major depressive disorder, recurrent episode
Diverticulosis	Codes that mention diverticulosis of small intestine or colon:<br>- ICD9 562.00 – Diverticulosis of small intestine<br>- ICD9 562.02 – Diverticulosis of small intestine with hemorrhage<br>- ICD9 562.10 – Diverticulosis of colon<br>- ICD9 562.12 – Diverticulosis of colon with hemorrhage<br>- ICD10 K57.x0 – Diverticulosis without bleeding<br>- ICD10 K57.x1 – Diverticulosis with bleeding
Diabetes mellitus, type 1	Codes that mention Diabetes Type 1:<br>- ICD9 250.x1 (excluding 250.01) – Diabetes mellitus, Type I, controlled<br>- ICD 9 250.x3 (excluding 250.03) Diabetes mellitus, Type I, uncontrolled<br>- ICD10 E10.x – Type 1 Diabetes mellitus
Diabetes mellitus, type 2, with complications	Codes that mention Diabetes Type 2 with any type of complication or uncontrolled:<br>- ICD9 249.x (excluding 249.00 and 249.01) – Secondary diabetes mellitus, with complications<br>- ICD9 250.x0 (excluding 250.00) – Diabetes mellitus, Type II, controlled<br>- ICD9 250.x2 (excluding 250.02) – Diabetes mellitus, Type II, uncontrolled<br>- ICD10 E08.x (excluding E08.9) – Diabetes mellitus<br>- ICD10 E09.x – Drug or chemical induced Diabetes mellitus<br>- ICD10 E11.x (excluding E11.9) – Type 2 Diabetes Mellitus<br>- ICD10 E13.x (excluding E13.9) – Other Specified Diabetes Mellitus
Diabetes mellitus, type 2, without complications	Codes that mention Diabetes Type 2 and specifically no complication:<br>- ICD9 249.0x – Secondary diabetes mellitus without mention of complication<br>- ICD9 250.0x – Diabetes mellitus without mention of complication<br>- ICD10 E08.9 – Diabetes mellitus due to underlying condition without complications<br>- ICD10 E11.9 – Type 2 diabetes mellitus without complications

	- ICD10 E13.9 – Other specified diabetes mellitus without complications
Epilepsy	Codes that mention epilepsy or recurrent seizures:<br>- ICD9 345.x Epilepsy and recurrent seizures<br>- ICD10 G40.x Epilepsy and recurrent seizures<br>- ICD10 Z82.0 Family history of epilepsy and other diseases of the nervous system<br>- ICD9 649.4x Epilepsy complicating pregnancy, childbirth, or the puerperium
End stage renal disease	Codes that mention stage V chronic kidney disease, end state renal disease, dialysis, or anything related to dialysis:<br>- ICD9 458.21 – Hypotension of hemodialysis<br>- ICD9 585.5 – CKD, Stage V<br>- ICD9 585.6 – ESRD<br>- ICD9 996.68 – Infection due to peritoneal dialysis catheter<br>- ICD9 996.73 – Complications due to renal dialysis<br>- ICD9 V45.1 – Postsurgical renal dialysis status<br>- ICD9 V45.11 – Renal dialysis status<br>- ICD9 V45.12– Noncompliance with renal dialysis<br>- ICD9 V56.x – Renal Dialysis Encounter<br>- ICD10 N18.5 – CKD, Stage V<br>- ICD10 N18.6 – ESRD<br>- ICD10 I13.11 – Hypertensive heart and CKD without heart failure , with ESRD<br>- ICD10 I13.2 - Hypertensive heart and CKD with heart failure , with ESRD<br>- ICD10 I12.0 – Hypertensive chronic kidney disease with stage 5 chronic kidney disease<br>- ICD10 I95.3 – Hypotension of hemodialysis<br>- ICD10 T85.611x – Breakdown of intraperitoneal dialysis catheter<br>- ICD10 T85.621A – Displacement of intraperitoneal dialysis catheter<br>- ICD10 T85.691A – Other mech complication of intraperitoneal dialysis catheter<br>- ICD10 T85.71XD – Infection and inflammatory reaction due to peritoneal dialysis catheter<br>- ICD10 T82.41Xx – Breakdown of vascular dialysis catheter<br>- ICD10:T82.43Xx – Leakage of vascular dialysis catheter<br>- ICD10 T82.49Xx – Other complications of vascular catheter<br>- ICD10 Y62.2 – Failure of sterile precautions during kidney dialysis and other perfusion<br>- ICD10 Y84.1 – Kidney dialysis as the cause of abnormal reaction of the patient<br>- ICD10 Z49.01 – Encounter for fitting and adjustment of extracorporeal dialysis catheter<br>- ICD10 Z49.02 - Encounter for fitting and adjustment of peritoneal dialysis catheter<br>- ICD10 Z49.3 – Encounter for adequacy testing for hemodialysis

	- ICD10 Z91.15 – Patient's noncompliance with renal dialysis<br>- ICD10 Z99.2 – Dependence on renal dialysis<br>Codes that include the medications epoetin alfa or bicarbonate hemodialysis solution with K, Ca, Mg (examples include):<br>- ERX 122830<br>- LMA 3772<br>- OMA BASX6
Gastroesophageal reflux disease	Codes that mention esophageal or gastroesophageal reflex:<br>- ICD9 530.11: Esophageal reflux<br>- ICD10 K21.x: Gastro-esophageal reflux disease
Gastrointestinal bleed	Codes that mention gastroesphageal bleed, hematemesis, or melena:<br>- ICD9 578.x: Gastrointestinal hemorrhage<br>- ICD10 K92.0: Hematemesis<br>- ICD10 K92.1: Melena<br>- ICD10 K92.2: Gastrointestinal Bleed, unspecified
Gout	Codes that mention gout:<br>- ICD9 274.x Gout<br>- ICD10 M10.x Gout<br>- ICD10 M1A.x Chronic Gout
Heart failure	Codes that mention any types of heart failure including systolic, diastolic, and rheumatic heart failure.<br>- ICD9 428.3x – Diastolic Heart failure<br>- ICD9: 428.4x – Combined systolic and diastolic heart failure<br>- ICD10 I50.3x – Diastolic heart failure<br>- ICD10 I50.4x – Combined systolic and diastolic heart failure<br>- ODA MLFT5 Diastolic heart failure<br>- ICD9 - 398.91 Rheumatic heart failure<br>- ICD9 402.11 Benign hypertensive heart disease with heart failure<br>- ICD9 402.91 Unspecified hypertensive heart disease with heart failure.<br>- ICD9 404.01 Hypertensive heart and chronic kidney disease, malignant, with heart failure and with chronic kidney disease stage I through stage IV, or unspecified<br>- ICD9 404.11 Hypertensive heart and CKD with heart failure and stage 1 through 4 chronic kidney disease, or unspecified<br>- ICD9 404.13 Hypertensive heart and CKD with heart failure and stage 5 CKD, or unspecified<br>- ICD9 404.91 Hypertensive heart and CKD with heart failure and with CKD I through IV<br>- ICD9 404.93 Hypertensive heart and CKD with heart failure and with CKD 5<br>- ICD9 428.0 Congestive heart failure<br>- ICD9 428.1 Left heart failure<br>- ICD9 428.2x – Systolic Heart Failure<br>- ICD9 428.4x – Combined systolic and diastolic heart failure

	- ICD10 I09.81 – Rheumatic heart failure<br>- ICD10 I13.0 – Hypertensive heart and chronic kidney disease with heart failure and stage 1 through 4 chronic kidney disease, or unspecified chronic kidney disease<br>- ICD10 I11.0 – Hypertensive heart disease with heart failure<br>- ICD10 I50.2x – Systolic (congestive) heart failure<br>- ICD10 I50.82 – Biventricular heart failure<br>- ICD10 I50.84 – End stage heart failure<br>- ICD10 I50.89 – Other heart failure<br>- ICD10 I50.4x – Combined systolic and diastolic heart failure<br>- ICD10 I50.9 - Heart failure, unspecified
Hyperlipidemia	Codes that include high cholesterol, disorders of lipid metabolism, and hyperlipidemia:<br>- ICD9 272.x – Disorders of lipoid metabolism<br>- ICD10 E78.x – Disorders of lipoprotein metabolism and other lipidemias
Hypertension	Codes that include hypertension:<br>- ICD 9 401.x – Essential Hypertension (excluding 401.0);-<br>- ICD 9 405.x – Secondary Hypertension (excluding 405.01 & 405.09);<br>- ICD10 I10.x Essential Hypertension;<br>- ICD10 O10.x – Pre-existing hypertension complicating pregnancy, childbirth, and the puerperium;<br>- ICD 10 O11.x Pre-existing hypertension with pre-eclampsia;<br>- ICD 9 642.13 Hypertension secondary to renal disease, complicating pregnancy, childbirth, and puerperium;<br>- ICD 9 997.91 - Complications affecting other specified body systems, not elsewhere classified, hypertension;<br>Codes that includes the medications hydrochlorothiazide and amlodipine. Of note many other medications for hypertension were not included since they are used for other diseases including congestive heart failure and chronic kidney disease and would not be specific for hypertension (examples include):<br>- ERX 100141<br>- LMA 953<br>- OMA MTDH2
Hypertensive emergency	Codes that include malignant hypertension or hypertensive emergency:<br>- ICD9 401.0 - Malignant hypertension<br>- ICD9 405.01 - Malignant renovascular hypertension<br>- ICD9 405.09 - Other malignant secondary hypertension<br>- ICD10 I16.x - Hypertensive Urgency and Emergency
History of pneumonia	Codes that include any type of viral, bacterial, or fungal pneumonia:<br>- ICD9 011.65 Tuberculosis Pneumonia<br>- ICD9 480.x Viral Pneumonia<br>- ICD9 481 Pneumococcal pneumonia<br>- ICD9 482.x Other bacterial pneumonia<br>- ICD9 483.x Pneumonia due to other specified organism<br>- ICD9 484.x Pneumonia in infectious diseases classified elsewhere

	- ICD9 485 Bronchopneumonia, organism unspecified<br>- ICD9 486 Pneumonia, organism unspecified<br>- ICD9 487.0 Influenza with pneumonia<br>- ICD9 488.11 Influenza due to identified 2009 H1N1 influenza with pneumonia<br>- ICD9 997.31 Ventilator associated pneumonia<br>- ICD10 B01.2 Varicella pneumonia<br>- ICD10 J09.X1 – Influenza due to identified novel influenza A virus with pneumonia<br>- ICD10 J10.0x – Influenza due to other identified influenza virus with pneumonia<br>- ICD10 J11.0x - Influenza due to unidentified influenza virus with pneumonia<br>- ICD10 J12.x – Other viral pneumonia<br>- ICD10 J13 – Pneumonia due to Strep pneumonia<br>- ICD10 J14 – Pneumonia due to H flu<br>- ICD10 J15.x – Bacterial Pneumonia<br>- ICD10 J16.x – Pneumonia due to other infectious organisms, not elsewhere classified<br>- ICD10 J17 Pneumonia in diseases classified elsewhere<br>- ICD10 J18.x Pneumonia, unspecified organism<br>- ICD10 J85.1 Abscess of lung with pneumonia<br>- ICD10 J95.851 Ventilator associated pneumonia<br>- ICD10 V1261 Pneumonia (recurrent)<br>- ICD10 Z87.01 Personal history of pneumonia (recurrent)
History of a urinary tract infection	Codes that include a urinary tract infection:<br>- ICD9 - 599.0 – Urinary tract infection, site not specified<br>- ICD10 - N39.0 – Urinary tract infection, site not specified<br>- ICD10 - O86.20 - Urinary tract infection following delivery
Hyperparathyroidism	Codes that include hyperparathyroidism, including both primary and secondary:<br>- ICD9 252.0x Hyperparathyroidism<br>- ICD9 588.81 Secondary hyperparathyroidism<br>- ICD10 E21.0- E21.3 Hyperparathyroidism<br>- ICD10 N25.81 Secondary hyperparathyroidism of renal origin
Hypothyroidism	Codes that include hypothyroidism, all types:<br>- ICD9 243 – Congenital hypothyroidism<br>- ICD9 244.x – Acquired hypothyroidism<br>- ICD10 E02 – Subclinical iodine deficiency hypothyroidism<br>- ICD10 E03.x – Other hypothyroidism<br>- ICD10 E89.0 – Postprocedural hypothyroidism<br>Codes that mention the medication levothyroxine (examples include):<br>- ERX 4420<br>- LMA 415<br>- OMA ETB21
Interstitial pulmonary disease	Codes that include interstitial pulmonary disease:<br>- ICD9 516.x – Other alveolar and parietoalveolar pneumonopathy

	- ICD10 J84.x – Other interstitial pulmonary disease
Mitral valve disorder	Codes that include mitral valve disorders, including insufficiency, stenosis, and regurgitation:<br>- ICD9 424.0 – Mitral Valve Disorders<br>- ICD10 I34.x – Mitral Valve Disorders
Breast neoplasm	Codes that include neoplasm of the breast or breast cancer:<br>- ICD9 174.x – Malignant Neoplasm of the Breast<br>- ICD10 C50.x - Malignant Neoplasm of the Breast
Lung neoplasm	Codes that include neoplasm of lung or lung cancer:<br>- ICD9 162.x (excluding where x = 0) Malignant Neoplasm of Bronchus and Lung<br>- ICD10 C34.x – Malignant Neoplasm of Bronchus and Lung
Prostate neoplasm	Codes that include malignant neoplasm of the prostate or prostate cancer:<br>- ICD9 185.x – Malignant Neoplasm of Prostate<br>- ICD10 C61.x – Malignant Neoplasm of Prostate
Osteoarthritis	Codes that include osteoarthrosis and osteoarthritis:<br>- ICD9 715.x – Osteoarthrosis and allied disorders<br>- ICD10 M15.x – Polyosteoarthritis<br>- ICD10 M16.x – Osteoarthritis of hip<br>- ICD10 M17.x – Osteoarthritis of knee<br>- ICD10 M18.x – Osteoarthritis of first carpometacarpal joint<br>- ICD10 M19.x – Other and unspecified osteoarthritis
Occlusion of the carotid artery	Codes that include occlusion of the carotid or stenosis carotid artery:<br>- ICD9 433.1 – Occlusion of Carotid Artery<br>- ICD10 I65.2x - Occlusion of Carotid Artery
Obstructive sleep apnea	Codes that include obstructive sleep disorder:<br>- ICD9 780.57 Unspecified sleep apnea<br>- ICD9 780.53 Hypersomnia with sleep apnea, unspecified<br>- ICD9 327.23 Obstructive Sleep Apnea<br>- ICD10 G47.33 Obstructive Sleep Apnea
Parkinson's disease	Codes that mention Parkinson’s Disease:<br>- ICD9 332.x – Parkinson’s Disease<br>- ICD10 G21.x – Parkinson’s Disease
Pulmonary embolism or Deep Venous Thrombosis	Codes that mention pulmonary embolism, atheroembolism, and thrombosis:<br>- ICD9 415.x – Acute pulmonary heart disease<br>- ICD9 453.x – Other venous embolism and thrombosis<br>- ICD9 445.x – Atheroembolism<br>- ICD9 673.x – Obstetrical pulmonary embolism<br>- ICD10 I26.x – Pulmonary embolism<br>- ICD10 I74.x – Arterial embolism and thrombosis<br>- ICD10 I75.x – Atheroembolism of unspecified lower extremity<br>- ICD10 I82.x – Other venous embolism and thrombosis<br>- ICD10 O88.x – Obstetric embolism<br>- ICD10 Z86.711 – Personal history of pulmonary embolism<br>- ICD10 Z86.718 – Personal history of other venous thrombosis and embolism

Pulmonary hypertension	Codes that mention primary hypertension:<br>- ICD9 416.0 – Primary pulmonary hypertension<br>- ICD10 I27.x Pulmonary Hypertension
Peripheral vascular disease	Codes that mention peripheral vascular disease:<br>- ICD9 443.9 – Peripheral vascular disease, unspecified<br>- ICD10 I73.9 – Peripheral vascular disease, unspecified
Rheumatoid arthritis	Codes the mention rheumatoid arthritis:<br>- ICD9 714.x Rheumatoid arthritis and other inflammatory polyarthropathies<br>- ICD10 M05.x Rheumatoid arthritis with rheumatoid factor<br>- ICD10 M06.x Other rheumatoid arthritis<br>- ICD10 M08.x Unspecified juvenile rheumatoid arthritis
Smoking history	Codes that mention tobacco use disorder:<br>- ICD9 305.1 Tobacco use disorder<br>- ICD9 305.1 Tobacco use disorder<br>- ICD10 Z71.6 – Tobacco abuse counseling<br>- ICD10 Z72.0 – Tobacco use
Thoracic aortic aneurysm	Codes that mention thoracic aneurysm, ruptured and unruptured:<br>- ICD9 441.1 – Thoracic aortic aneurysm, ruptured<br>- ICD9 441.2 – Thoracic aortic aneurysm without mention of rupture<br>- ICD10 I71.1 – Thoracic aortic aneurysm, ruptured<br>- ICD10 I71.2 – Thoracic aortic aneurysm, without rupture
Tricuspid valve disorder	Codes that mention tricuspid valve disorder, including insufficiency, regurgitation, and stenosis:<br>- ICD9 397.0 – Diseases of tricuspid valve<br>- ICD10 I36.x – Tricuspid valve disorder
Vitamin D deficiency	Codes that mention a Vitamin D disorder:<br>- ICD9 268.9 – Unspecified Vitamin D deficiency<br>- ICD10 E55.9 – Vitamin D deficiency
Ventricular tachycardia	Codes that mention a ventricular tachycardia:<br>- ICD9 427.1 – Paroxysmal Ventricular Tachycardia<br>- ICD10 I47.2 – Ventricular Tachycardia

Supplementary Table 2. GLM Boost Feature Odds Ratios Across Models

** Odds Ratio values are the median over 10 model iterations.*

*** Features are ordered by median Odds Ratio for each model.*

Overall model

Feature	Odds Ratio	IQR	seen in	% with condition
Age	2.816	0.052	10	100%
History of pneumonia	1.060	0.016	10	20%
Diabetes mellitus, type 2, with complications	1.047	0.015	10	12%
Heart failure	1.047	0.011	10	8%
Chronic kidney disease	1.042	0.014	10	9%
Interstitial pulmonary disease	1.027	0.025	5	2%
Chronic obstructive pulmonary disease	1.024	0.021	10	5%
Pulmonary embolism	1.018	0.019	8	6%
Benign prostate hypertrophy	1.016	0.019	6	11%
Atrial fibrillation and flutter	1.016	0.003	4	8%
Hypertensive urgency or emergency	1.015	0.021	8	2%
Coronary artery disease	1.014	0.003	2	12%
Gout	1.012	0.006	3	4%
Lung neoplasm	1.012	0.000	2	1%
History of a cerebrovascular accident	1.009	0.003	2	8%
Abdominal aortic aneurysm	1.006	0.000	2	1%
Cardiomegaly	1.006	0.000	2	5%
Female	0.972	0.017	9	57%

45-65 Model

Feature	Odds Ratio	IQR	seen in
Age	1.189	0.062	10
Diabetes mellitus, type 2, with complications	1.164	0.030	10
Gastrointestinal bleed	1.082	0.019	10
Tricuspid valve disorder	1.073	0.015	2
Breast cancer	1.069	0.032	8
Atrial fibrillation and flutter	1.043	0.039	9
Pulmonary embolism	1.036	0.023	10
History of pneumonia	1.032	0.049	8
Chronic kidney disease	1.028	0.008	5
History of a cerebrovascular accident	1.028	0.014	6
Prostate neoplasm	1.026	0.010	6
Heart failure	1.026	0.012	5
Ventricular tachycardia	1.021	0.017	2

Coronary artery disease	1.020	0.011	3
Hypertensive urgency or emergency	1.019	0.016	5
White	1.009	0.000	2
Aortic valve disorder	1.008	0.002	2
Female	0.917	0.030	8

65-85 Model

Feature	Odds Ratio	IQR	seen in
Age	1.361	0.079	10
History of pneumonia	1.130	0.027	10
Chronic kidney disease	1.119	0.041	10
Interstitial pulmonary disease	1.081	0.052	10
Smoker	1.064	0.040	9
Heart failure	1.054	0.022	9
Pulmonary embolism	1.042	0.024	9
Hypertensive urgency or emergency	1.042	0.036	8
Chronic obstructive pulmonary disease	1.038	0.066	10
Cardiomegaly	1.036	0.030	8
End stage renal disease	1.035	0.029	6
Occlusion of the carotid artery	1.031	0.017	5
Black	1.030	0.010	2
Lung neoplasm	1.028	0.019	6
Anemia	1.027	0.006	8
Hyperparathyroidism	1.020	0.032	8
Diabetes mellitus, type 2, without complications	1.014	0.009	4
Abdominal aortic aneurysm	1.014	0.014	7
Rheumatoid arthritis	1.014	0.012	8
Gout	1.009	0.002	2
Atrial fibrillation and flutter	1.009	0.014	2
Female	0.905	0.024	10

85+ Model

Feature	Odds Ratio	IQR	seen in
Chronic kidney disease	1.097	0.041	10
Hypertensive urgency or emergency	1.094	0.013	9
Diabetes mellitus, type 2, with complications	1.067	0.027	3
History of a cerebrovascular accident	1.063	0.027	9
Age	1.061	0.025	10
Anemia	1.051	0.033	10

Cardiomegaly	1.048	0.014	8
Diabetes mellitus, type 2, without complications	1.047	0.036	7
Benign prostate hypertrophy	1.046	0.053	9
Pulmonary embolism	1.044	0.034	9
Vitamin D deficiency	1.037	0.030	7
Rheumatoid arthritis	1.028	0.001	3
Ventricular tachycardia	1.020	0.031	8
Abdominal aortic aneurysm	1.019	0.023	4
Black	1.018	0.004	3
lung neoplasm	1.013	0.017	6
Chronic obstructive pulmonary disease	1.007	0.000	2
White	0.993	0.000	2
Hispanic	0.914	0.044	10
Female	0.878	0.037	10
